# Combination treatment strategy for pancreatic cancer involving the novel HDAC inhibitor MPT0E028 with a MEK inhibitor beyond K-Ras status

**DOI:** 10.1186/s13148-019-0681-6

**Published:** 2019-05-29

**Authors:** Min-Wu Chao, Li-Hsun Chang, Huang-Ju Tu, Chao-Di Chang, Mei-Jung Lai, Yi-Ying Chen, Jing-Ping Liou, Che-Ming Teng, Shiow-Lin Pan

**Affiliations:** 1 Graduate Institute of Cancer Molecular Biology and Drug Discovery, College of Medical Science and Technology, Taipei Medical University, Taipei, Taiwan; 2 Pharmacological Institute, College of Medicine, National Taiwan University, Taipei, Taiwan; 3 Ph.D. Program in Biotechnology Research and Development, College of Pharmacy, Taipei Medical University, Taipei, Taiwan; 4 School of Pharmacy, College of Pharmacy, Taipei Medical University, Taipei, Taiwan; 5 Biomedical Commercialization Center, Taipei Medical University, Taipei, Taiwan; 6 The Research Center of Cancer Translational Medicine, Taipei Medical University, Taipei, Taiwan

**Keywords:** Pancreatic cancer, MPT0E028, MEK inhibitor, EGFR, K-Ras status

## Abstract

**Background:**

Oncogenic K-Ras signaling highly relies on the canonical Ras/MEK/ERK pathway to contribute to pancreatic cancer progression. However, numerous efforts of MEK inhibitors have failed to provide an optimal antitumor effect for pancreatic cancer in practice. The aim of the present work was to develop a more efficacious therapeutic intervention for MEK inhibitors through combination with histone deacetylase (HDAC) inhibitor MPT0E028.

**Methods:**

The effects of combined therapy on cell viability, apoptosis, protein, and RNA expressions were determined by MTT assay, flow cytometry, western blotting, and quantitative PCR analysis. The AsPC-1 xenograft was used to assess antitumor effects in vivo*.*

**Results:**

The co-administration of MPT0E028 and MEK inhibitor yielded synergistic effects on cell viability suppression both in K-Ras mutated and wild-type pancreatic cancer cells and also markedly triggered cell apoptosis. Surprisingly, ERK and epidermal growth factor receptor (EGFR) were activated by the long-term and low-concentration treatment of MPT0E028 or another HDAC inhibitor alone. Whereas, the pharmacological attenuation of ERK signaling dramatically abolished the MPTE028-induced p-ERK and EGFR expression. Overexpression of HDAC4, HDAC6, and MEK, respectively, reversed the cell death induced by the combined treatment. Finally, the combined treatment decreased the tumor volume in an AsPC-1 xenograft model compared to each individual treatment alone.

**Conclusions:**

The synergistic anti-survival effect of the combination was suggested to occur via compensation of the MEK inhibitor for activated ERK. Our results indicate that this combination strategy could benefit patients with pancreatic cancer beyond K-Ras status.

**Electronic supplementary material:**

The online version of this article (10.1186/s13148-019-0681-6) contains supplementary material, which is available to authorized users.

## Background

Pancreatic cancer accounts for 3% of all cancers but remains the fourth leading cause of cancer-related deaths. The overall 5-year survival rate for patients with pancreatic cancer is less than 5% [1]. Compounding this poor prognosis is the limited therapeutic options against pancreatic cancer. Gemcitabine is used as a standard chemotherapy but only improves overall survival by 5.7 months, while the combination of gemcitabine with the targeted therapy erlotinib only enhances overall survival by 6.24 months. Recently, the combination of 5-fluorouracil, irinotecan, and oxaliplatin was shown to provide a significant increase of the overall survival rate (11.1 months) for patients with pancreatic cancer but was also associated with a high toxicity rate [2]. Therefore, development of a new therapeutic strategy for pancreatic cancer is an urgent clinical challenge.

Mutations of oncogenic genes are largely associated with triggering cancer cell proliferation, metastasis, and angiogenesis. K-Ras mutations are present in approximately 95% of pancreatic cancers, leading to burst activation of the mitogen-activated protein kinase (MAPK) pathway, also known as the Ras-Raf-MEK-ERK pathway [3]. In transgenic mouse models, K-Ras mutation has been shown to play an early oncogenic role in the development and progression of pancreatic cancer. Furthermore, cooperation of the Ras-Raf-MEK-ERK pathway with other genetic alterations, including *p16*^*INK4a*^, *p53*, or *TGFβ/SMAD4*, accelerates pancreatic cancer progression [4, 5]. Activation of the Ras-Raf-MEK-ERK pathway promotes cell cycle progression through increasing cyclin D expression and inhibits apoptosis by repressing the pro-apoptotic Bcl-2 family of proteins [6, 7]. Thus, targeting the MAPK pathway appears to be a valuable strategy for the treatment of pancreatic cancer. However, few MEK inhibitors have been developed and applied in clinical trials for pancreatic cancer to date. CI-1040, developed by Pfizer/Warner-Lambert, was the first MEK inhibitor that progressed to the clinical stage of evaluation but failed in a phase II trial due to its poor exposure (ClinicalTrials.gov number, NCT00033384) [8, 9]. Another MEK inhibitor, selumetinib (AZD6244, AstraZeneca), showed no superior effect in pancreatic cancer patients when compared with conventional cytotoxic chemotherapy [10]. Thus, one potential strategy to overcome these issues is to develop a combination treatment of a MEK inhibitor with other anticancer agents, which requires further investigation.

Recent research demonstrated that some tumor suppression genes are epigenetically silenced in cancers. Histone deacetylases (HDACs) and histone acetyltransferases are two important enzymes that regulate the post-translational modification of histones and consequent gene expression [11]. HDACs have long been recognized as transcriptional repressors and are found to be overexpressed in several types of cancer, including pancreatic cancer [12]. Blocking HDAC activities with HDAC inhibitors such as vorinostat (SAHA) or trichostatin A results in a potent antitumor effect, especially in hematologic malignancies. Treatment of HDAC inhibitors was shown to directly promote the apoptosis pathway through induction of proapoptotic genes (*BMF*, *BIM*), generation of reactive oxygen species, and upregulation of TRAIL expression [13]. In addition, the combination of HDAC inhibitors and chemotherapies has a synergistic effect on antitumor activity. For instance, co-treatment of an HDAC inhibitor plus gemcitabine synergistically enhanced apoptosis and the cytotoxic effect of each agent in pancreatic cancer cells [14].

MPT0E028 is a novel pan-HDAC inhibitor, which targets both classes I and II HDAC, with potent antitumor activity demonstrated not only in hematologic cancers but also in solid tumors [15, 16]. A previous study indicated that MPT0E028 showed more potent effects for inhibiting HDAC activity and inducing cell apoptosis compared to SAHA both in vitro and in vivo [15]. A phase I trial of MPT0E028 is currently completed in March of 2019 (ClinicalTrials.gov number, NCT02350868). Given that a K-Ras mutation is a key mechanism of pancreatic carcinogenesis, we hypothesized that treatment of MEK inhibitors could interrupt the K-Ras downstream signaling pathway to reduce the survival of pancreatic cancer cells. Although several studies have demonstrated that combination of an HDAC inhibitor with a MEK inhibitor had synergistic antitumor activity [17–19], the effect and underlying mechanism of this combinational strategy for pancreatic cancer have yet to be explored in detail. Accordingly, the objective of the present study was to exploit the antitumor activity of the combination of the novel HDAC inhibitor MPT0E028 with a MEK inhibitor, and determine the feasibility of this strategy for improving the therapeutic outcome in the treatment of pancreatic cancer.

## Methods

### Reagents

RPMI-1640 medium, DMEM medium, fetal bovine serum (FBS), penicillin, streptomycin, and all other tissue culture reagents were obtained from GIBCO/BRL Life Technologies (Grand Island, NY, USA). MPT0E028 was synthesized by our chemical team. Briefly, the commercially available 1H-indole-5-carbaldehyde with benzenesulfonyl chloride yielded the related 1-benzenesulfonylindole. This compound was subject to the Wittig reaction with methyl (triphenylphosphorylidene) acetate followed by LiOH hydrolysis and PYBOP-mediated amide formation, and the reaction sequence was completed by TFA-mediated deprotection to afford the desired 1-benzenesulfonyl-5-(*N*-hydroxyacrylamide)-indole, MPT0E028 [20]. PD98059, propidium iodide (PI), and 3-(4,5-dimethylthiazol-2-yl)-2,5-diphenyltetrazolium bromide (MTT) were ordered from Sigma Chemical (St. Louis, MO, USA). Vorinostat (SAHA) and trametinib were purchased from Selleckchem (Houston, TX, USA). Trizol reagent and Lipofectamine 2000 were from Invitrogen (Carlsbad, California, USA); random primer and M-MLV RT were purchased from Promega (Madison, WI, USA). SYBR™ Green PCR Master Mix was from Applied Biosystems/Thermo Fisher Scientific (Waltham, Massachusetts, USA).

Actin antibody was purchased from Millipore (Burlington, MA, USA). Antibody against caspase-3 was purchased from Imgenex (San Diego, CA, USA). HRP-conjugated anti-mouse and anti-rabbit IgGs were ordered from Jackson ImmunoResearch (PA, USA). Antibodies specific to PARP, EGFR, phosphorylated (Thr-202/Tyr-204) or total p44/p42 MAP kinase, MEK, H3K9ac, GFP, HDAC1, HDAC4, and HDAC6 were obtained from Cell Signaling Technology (Beverly, MA, USA).

### Cell culture

Human pancreatic cancer cell lines AsPC-1, PANC-1, and BxPC-3 were all purchased from Bioresource Collection and Research Center (BCRC; Hsinchu, Taiwan). AsPC-1 and BxPC-3 cells were cultured in RPMI-1640, and PANC-1 cells were maintained in DMEM. Both mediums are with 10% FBS (*v*/*v*). Mediums were supplemented with 100 U mL^−1^ penicillin, 100 μg mL^−1^ streptomycin, and 2.5 μg/mL amphotericin B. Cells were maintained in a humidified incubator at 37 °C in 5% CO_2_/95% air.

### Cell viability assay

Cell viability was determined by MTT assay. Cells were seeded into 96-wells for overnight and then treated with indicated concentrations of drugs for 72 h, washed out once and incubated with medium contained 0.5 mg/mL MTT for 1 h. Cells were lysed by DMSO and then the absorbance was detected by an ELISA reader at 550 nm wavelength. Cell viability was calculated by the ratio (percentage) of absorbance between control and treatment groups. Combination index (CI) and fractional effect (Fa) were measured by CompuSyn software (ComboSyn, Inc., Paramus, NJ, USA).

### Flow cytometry analysis

Cells were seeded into 6-wells for overnight. After adherence, cells were treated with indicated concentrations of drugs for 72 h and then collected by trypsinization, fixed with 75% (*v*/*v*) ethanol at − 20 °C overnight. After centrifugation, cells were incubated in phosphate-citric acid buffer (NaHPO_4_ 0.2 M, citric acid 0.1 M (pH 7.8)) for 20 min at room temperature. Then, cells were centrifuged and resuspended with 0.5 mL PI solution (Triton X-100 0.1%, RNase 100 μg/mL, and propidium iodide 80 μg/mL). DNA content was analyzed with the FACScan and CellQuest software (Becton Dickinson).

### Western blot analysis

Cell lysates were extracted by a lysis buffer containing Tris 50 mM, sodium chloride 150 mM, SDS 0.1%, sodium deoxycholate 0.5%, and NP-40 1% for 30 min at 4 °C and then centrifuged at 13,000 rpm at 4 °C for 30 min. Protein was quantified by BCA Protein Assay Kit (ThermoFisher Scientific, Waltham, Massachusetts, USA). Equal protein amounts of protein were resolved by 10% SDS-polyacrylamide gel and then transferred onto a nitrocellulose membrane after electrophoresis. The membranes were incubated with specific primary antibodies overnight at 4 °C and then applied to appropriate secondary antibodies for 1 h. The stock concentrations of the indicated primary antibodies were as following: PARP 433.0 μg/mL, caspase-3 1 mg/mL, H3K9ac 41.0 μg/mL, p-ERK 19.0 μg/ml, t-ERK 177.0 μg/mL, EFGR 29.0 μg/mL, HDAC1 241.0 μg/mL, HDAC4 320.0 μg/mL, HDAC6 46.0 μg/mL, GFP 7.0 μg/mL, and MEK 64.0 μg/mL. All the primary antibodies were used in 1:1000 dilution in TBST. After washing out the unbound primary antibodies, the membranes were applied to appropriate horseradish peroxidase-conjugated anti-mouse (for Caspase3, EGFR, HDAC1 and HDAC4, and MEK) or anti- rabbit immunoglobulin G secondary antibodies (for PARP, H3K9ac, p-ERK, t-ERK, HDAC6, and GFP) with 1:5000 dilution in TBST for 1 h at room temperature. Secondary bound antibodies were detected using enhanced chemiluminescence (ECL) detection reagents (Amersham, England). Briefly, working HRP substrate is prepared, and the membranes were incubated in equal volume of solution A and B for 1 min. Then removing HRP substrate, the membranes were exposed to X-ray film. An X-ray film was immersed in the developer and then in the fixer (Kodak, Sigma-Aldrich), and the signals were detected.

### Apoptotic cell death

Apoptotic cell death was assessed by human active caspase-3 ser29 ELISA kit (Abcam, Cambridge, MA, USA). The steps were followed by the provided protocol booklet. Briefly, cells were treated with indicated concentrations of drugs for 24, 48, and 72 h. The cell lysates were collected, extracted with the lysis buffer, and then added to appropriate well. Primary antibody cocktail was added to each well for 1 h at room temperature with shaking. The wells were washed three times, added with TMB substrate, and incubated for 1 h at room temperature with shaking. Finally, stop solution was added for the detection signal at a defined endpoint. The absorbance was detected by an ELISA reader at 450 nm wavelength.

### Transient transfection

Cells were seeded into 6-wells for overnight and then transfected with HDAC1, HDAC4, HDAC6, or MEK plasmids and ERK siRNA by using Lipofectamine 2000 for 8 h. PcDNA-FLAG-HDAC1 (plasmid 13820), pcDNA-FLAG-HDAC4 (plasmid 13821), pcDNA-FLAG-HDAC6 (plasmid 13823), and GFP-MEK (plasmid 14746) were purchased from Addgene (Cambridge, MA, USA), and ERK siRNA (s11137) was purchased from Thermo Fisher Scientific (Waltham, Massachusetts, USA). Refreshed culture medium for overnight and then treated with MPT0E028, PD98059, or both drugs for 48 h. Cell lysates were collected for western blot analysis.

### Real-time polymerase chain reaction (qPCR)

Total RNA was isolated with TRIzol reagent by a procedure protocol. According to the manufacturer’s protocol, 5 μg messenger RNA (mRNA) was incubated with random primer at 65 °C for 5 min and then reacted with M-MLV RT (Reverse Transcriptase) at 37 °C for 1 h to obtain cDNA. The SYBR™ Green PCR Master Mix (Applied Biosystems) was used to evaluate the amplification of EGFR and 18S housekeeper gene. EGFR and 18S primer sequences are 5′-GCGTCTCTTGCCGGAATG-3′/5′-CTTGGCTCACCCTCCAGAAG-3′ and 5′-AACCCGTTGAACCCCATT-3′/5′-CCATCCAATCGGTAGTAGC-3′, respectively. StepOne Real-Time PCR System (Applied Biosystems) was used for the detection of fluorescent signal. Relative fold gene changes were normalized to 18S and calculated by using the 2^−ΔΔCt^ method [21].

### In vivo xenograft animal model

Animal studies were reported in compliance with the ARRIVE guidelines [22, 23]. AsPC-1 xenograft animal model was referred and followed by the previous study [24, 25]. Four-week-old male Balb/c-nude mice were purchased from the National Laboratory Animal Center (Taipei, Taiwan). All mice were housed (five mice per cage) in specific pathogen-free (SPF) animal rooms with controlled temperature (20–22 °C) and humidity (60%) under a 12-h light/dark cycle. Before the start of experimentation, mice were offered ad libitum food and water for 7 days. AsPC-1 cells (1 × 10^7^/mice) were subcutaneously injected into Balb/c-nude mice to establish AsPC-1 xenograft model. Once tumor volume reached to 100 mm^3^, mice were divided in to four groups (*n* = 5) and dosed with vehicle (1% carboxymethyl cellulose + 0.5% Tween 80), PD98059 (20 mg/kg, ip (intraperitoneal), qd (once per day)), MPT0E028 (25 mg/kg, oral, qd), and PD98059 combined with MPT0E028 to the end of the experiment. Tumor volumes and body weight were measured twice per week. Tumor volumes were determined by the formula volume (mm^3^) = (length × width^2^)/2. The mice were scarified once tumor volume is above 1200 mm^3^. All protocols were followed and approved by the Animal Care and User Committee at the Taipei Medical University.

### Statistical analysis

The data and statistical analysis comply with the recommendations on experimental design and analysis [26]. In vitro experiments were obtained at least three independent times, and data are presented as means ± SD. Data statistical analysis was conducted by using Sigma Plot 10.0 and Prism 7.0 and evaluating with Student’s *t* tests for comparison of two groups. Prism 7.0 with Student’s *t* test was used for the analysis of animal study. Differences were considered significant at *P <* 0.05. One asterisk indicates *P* < 0.05, two asterisks indicate *P* < 0.01, and three asterisks indicate *P* < 0.001.

### Randomization and blinding

The animal study in this research was conducted in a randomized manner. The mice were randomly separated to cages by vivarium staff and randomized to vehicle or indicated treatment groups. During treatment, the investigator was blinded to each group. The operator and analyst were different people for blinding.

## Results

### MEK inhibitor potentiates the cytotoxic effect of MPT0E028 or SAHA in pancreatic cancer independently from K-Ras status

**Fig. 1:**
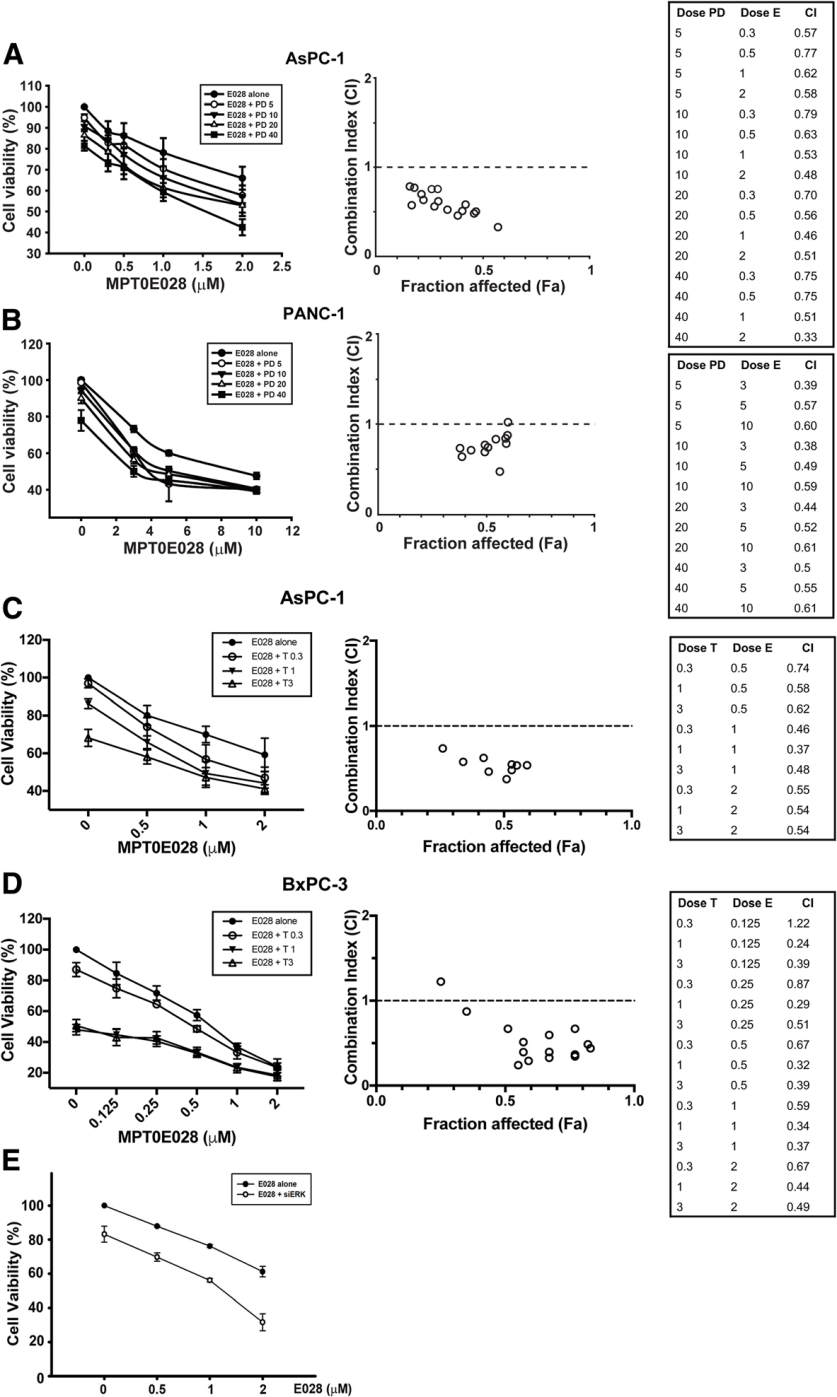
Cytotoxic effect of combination MPT0E028 with MEK inhibitors in pancreatic cancer cells. AsPC-1 (**a**) and PANC-1 (**b**) cells were treated with DMSO, MPT0E028 (E028), PD98059 (PD), or MPT0E028 plus PD98059 with indicated concentration for 72 h. AsPC-1 (**c**) and BxPC-3 (**d**) cells were treated with DMSO, MPT0E028, trametinib (T), or MPT0E028 plus trametinib with indicated concentration for 72 h. Left panels: Cell viability was determined by MTT assay. Right panels: Combination index (CI) and fraction affected (Fa) are calculated by CompuSyn software. **e** AsPC-1 cells were transfected with scramble or ERK siRNA and then combined with indicated concentrations of MPT0E028 for 48 h. PD 5, PD 10, PD 20, and PD 40 were represented here as PD98059 5 μM, 10 μM, 20 μM and 40 μM. T 0.3, T 1, and T 3 were represented here as trametinib 0.3 nM, 1 nM, and 3 nM

**Fig. 2:**
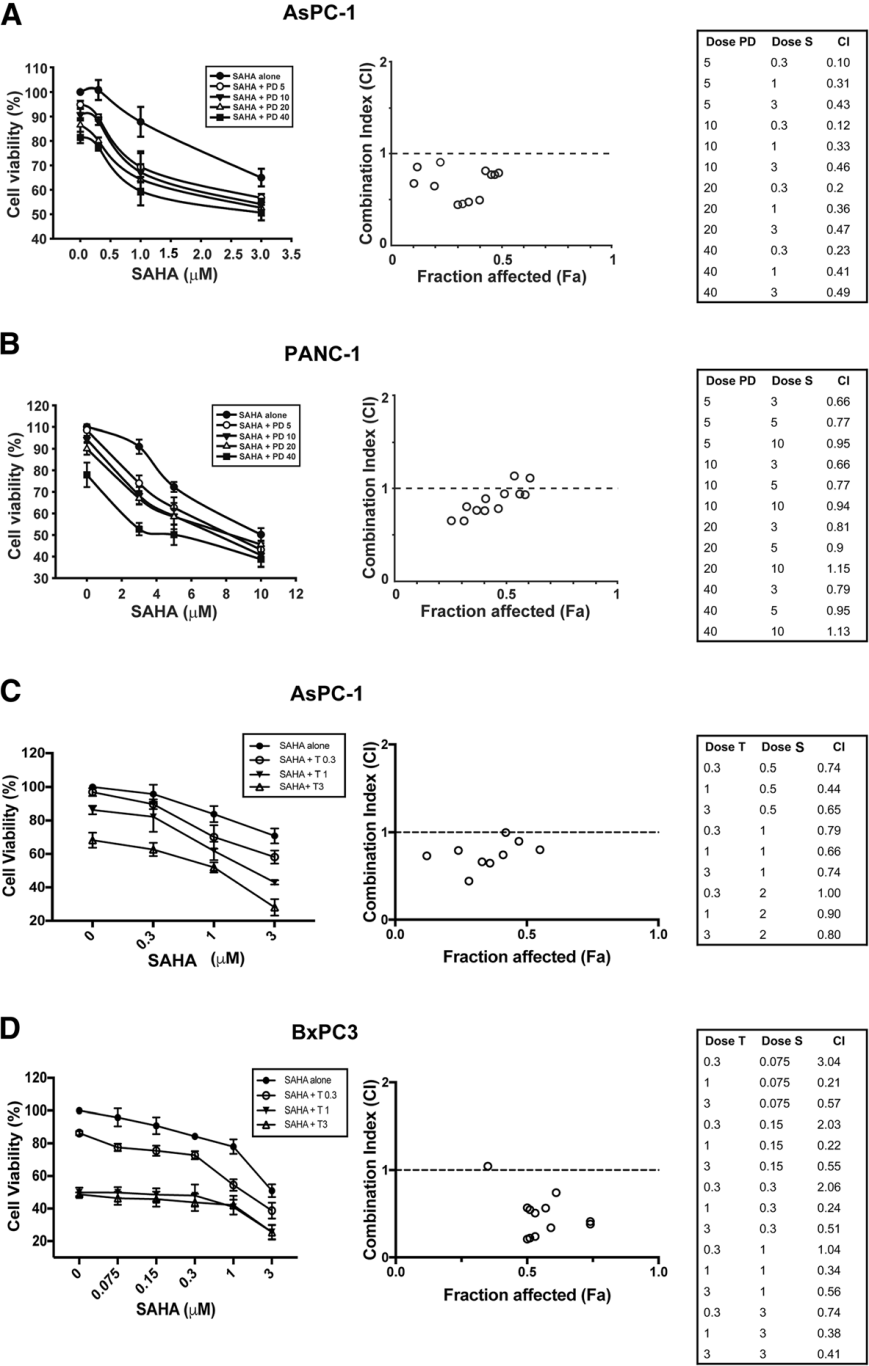
Cytotoxic effect of combination SAHA with MEK inhibitors in pancreatic cancer cells. AsPC-1 (**a**) and PANC-1 (**b**) cells were treated with DMSO, SAHA, PD98059 (PD), or SAHA plus PD98059 with indicated concentration for 72 h. AsPC-1 (**c**) and BxPC-3 (**d**) cells were treated with DMSO, SAHA, trametinib (T), or SAHA plus trametinib with indicated concentration for 72 h. Left panels: Cell viability was determined by MTT assay. Right panels: Combination index (CI) and fraction affected (Fa) are calculated by CompuSyn software. PD 5, PD 10, PD 20, and PD 40 were represented here as PD98059 5 μM, 10 μM, 20 μM and 40 μM.T 0.3, T 1, and T 3 were represented here as trametinib 0.3 nM, 1 nM, and 3 nM

First, we determined the cytotoxic effect of the HDAC inhibitor MPT0E028 or SAHA plus the selective MEK/ERK kinase inhibitor PD98059 or trametinib in three different pancreatic cancer cell lines. As shown in Additional file 5: Table S1, treatment with MPT0E028 or SAHA alone had a differentiated cytotoxic effect in all pancreatic cancer cells. The dosages of MPT0E028 and SAHA chosen for combination treatment were above or close to IC_50_ in each cell lines (Additional file 5: Table S1). The combination of MPT0E028 or SAHA with PD98059 displayed a synergistic cytotoxic effect in the K-Ras mutation-positive AsPC-1 and PANC-1 cell lines (Figs. 1a, b and 2a, b), and these phenomena also confirmed with trametinib, the only MEK inhibitor currently approved for the treatment of melanoma harboring a B-Raf mutation (Figs. 1c and 2c and Additional file 1: Figure S1C and D) [27]. Furthermore, the combined treatment also potentiated the effect of the HDAC inhibitor alone in BxPC-3 cells with wild type K-Ras (Figs. 1d, 2d and Additional file 1: Figure S1A and B). A combination index (CI) value of 1 indicates an additive drug interaction, whereas a CI value of less than 1 denotes synergism. To mimic the function of MEK inhibitor, we silenced MEK, showing a similar effect when the AsPC-1 cells were co-treated with MPT0E028 and PD98059 (Fig. 1e). Overall, these results showed that inhibition of MEK magnified the cytotoxic impact of pan-HDAC inhibitors both in K-Ras-mutated and wild-type pancreatic cancer cells.

### MPT0E028-mediated cell apoptosis is enhanced by MEK inhibitor

**Fig. 3:**
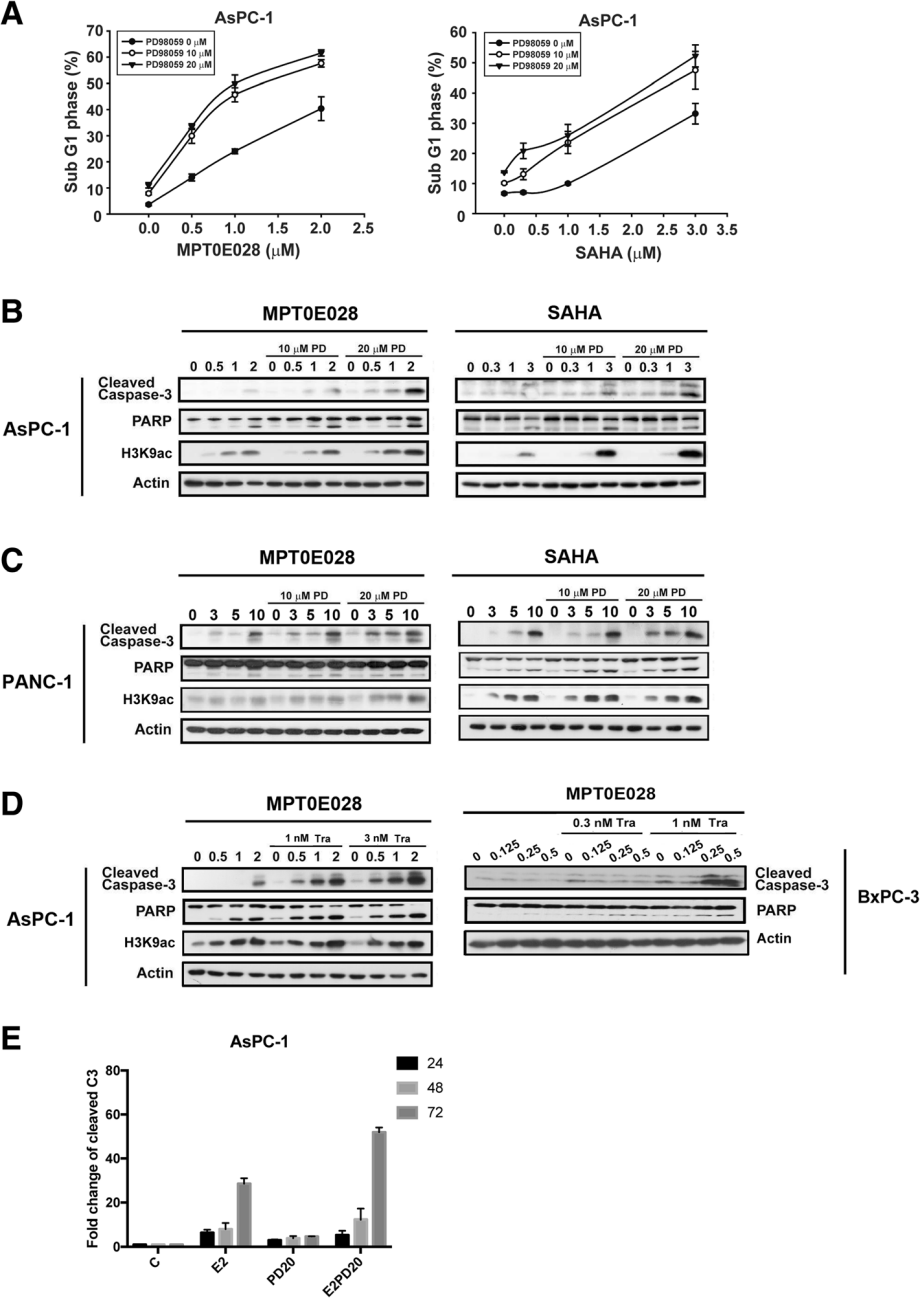
Combination HDAC inhibitors with MEK inhibitors enhance cell apoptosis in pancreatic cancer cells. **a** AsPC-1 cells were treated by MPT0E028 plus PD98059 (left panel) or SAHA plus PD98059 (right panel) with indicated concentrations for 72 h. sub-G1 cells were detected by flow cytometry and calculated by CellQuest software. AsPC-1 (**b**) and PANC-1 (**c**) cells were treated by DMSO, MPT0E028, MPT0E028 plus PD98059 (PD) or SAHA, and SAHA plus PD98059 with indicated concentrations for 72 h. AsPC-1 or BxPC-3 (**d**) cells were treated by DMSO, MPT0E028, and MPT0E028 plus trametinib (Tra) with indicated concentrations for 72 h. The protein level of cleaved caspase-3, PARP, H3K9ac, and actin were determined by western blotting. Actin was used as an internal control. **e** AsPC-1 cells were treated with DMSO, 2 μM MPT0E028 (E2), 20 μM PD98059 (PD20), and MPT0E028 plus PD98059 (E2PD20) for 24, 48, and 72 h. The fold change of cleaved caspase-3 was assessed by cleaved caspase-3 ELISA kit

To clarify the cytotoxic mechanism of the combined therapy, we firstly evaluated the percentage of cells in the sub-G1 phase of the cell cycle with a DNA content less than 2N after treatment. As demonstrated in Fig. 3a, PD98059 dose-dependently increased the proportion of AsPC-1 cells in the sub-G1 phase at 72 h after MPT0E028 or SAHA treatment. Since the cells with sub-G1 DNA content were counted as apoptotic and necrotic cells [28], further to focus on apoptosis, the cleaved caspase-3 induction was assessed. Treatment of AsPC-1 and PANC-1 cells with a high dose of MPT0E028 or SAHA alone resulted in caspase-3 cleavage and PARP activation, markers of induction of the apoptotic pathway. These effects were enhanced by adding PD98059 (Fig. 3b and c) or trametinib (Fig. 3d). The expression level of acetylated H3, an HDAC inhibitor biomarker, in PD98059 combined treatment was increased more significantly than observed with MPT0E028 or SAHA treatment alone (Fig. 3b and c), but not shown in the combination of trametinib (Fig. 3d). The differences between these results should be further investigated. Moreover, the combined treatment caused an apoptotic effect at 72 h was more significant than 48 h (Fig. 3e). To sum up, HDAC inhibitors caused greater apoptosis under MEK inhibition.

### MEK inhibitor compensated for MPT0E028-induced p-ERK and EGFR upregulation

**Fig. 4:**
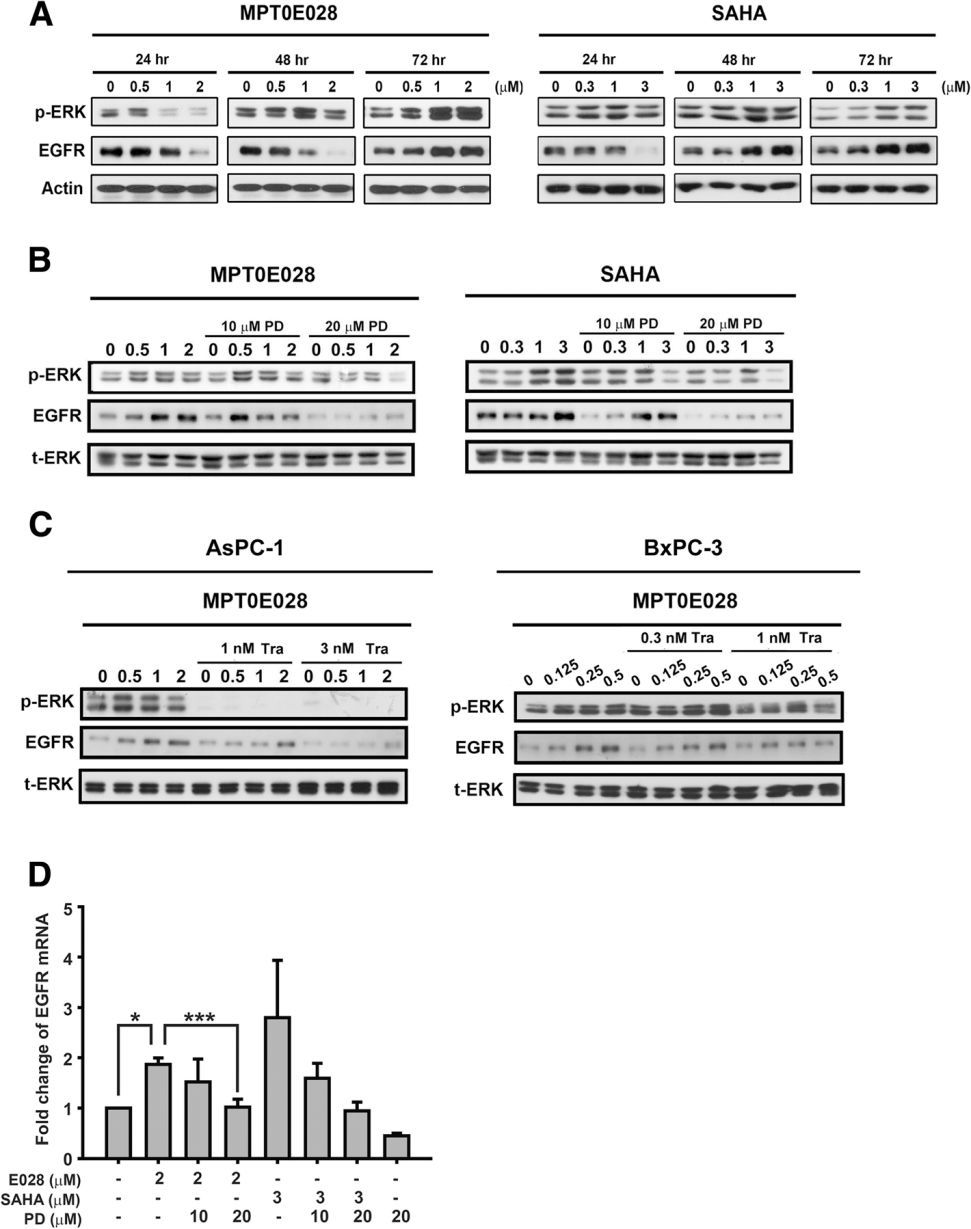
MEK inhibitors downregulate HDAC inhibitor-induced p-ERK and EGFR expression. **a** AsPC-1 cells were treated with indicated concentrations of MPT0E028 or SAHA for 24, 48, and 72 h. The protein expression of p-ERK and EGFR was determined by western blotting. Actin was used as an internal control. **b** AsPC-1 cells were treated with indicated concentrations of MPT0E028 or SAHA combined with or without 10 and 20 μM PD98059 (PD) for 72 h. **c** AsPC-1 and BxPC-3 cells were treated with indicated concentrations of MPT0E028 or combined with or without trametinib (Tra) for 72 h. p-ERK and EGFR protein expressions were analyzed. t-ERK was used as an internal control. **d** AsPC-1 cells were treated with indicated conditions for 48 h to determine EGFR mRNA expression. *P < 0.05 and ***P < 0.001 compared with the indicated group

To further elucidate the mechanisms underlying the synergistic action of this combined strategy, we treated the cells with MPT0E028 or SAHA over the long term and evaluated the effects on ERK activation. The levels of p-ERK and EGFR were reduced at 24 and 48 h with MPT0E028 and 24 h with SAHA treatment. However, each HDAC inhibitor alone triggered ERK phosphorylation and EGFR expression upregulation in a concentration-dependent manner in AsPC-1 cells at 72 h (Fig. 4a). And this MPT0E028 or SAHA-triggered p-ERK and EGFR upregulation could be eliminated by co-treatment with PD98059 or trametinib at 72 h in three pancreatic cells (Fig. 4b, c and Additional file 2: Figure S2A). Moreover, MPT0E028 or SAHA-regulated EGFR expression was found to occur through transcriptional activation at 48 h, and the effect was suppressed with PD98059 or trametinib treatment (Fig. 4d, Additional file 2: Figure S2B and C), which reflected to protein expression at 72 h (Fig. 4b, c and Additional file 2: Figure S2A ) in three pancreatic cells. Based on previous studies, K-Ras signaling activation occurs through the canonical MAPK pathway, which will be enhanced by positive feedback activation of EGFR [29]. Thus, we sought to clarify whether the expression of EGFR can be regulated by ERK activity. The results showed that PD98059 inhibited the EGFR protein level in a dose- and time-dependent manner (Additional file 3: Figure S3A), and the effect was confirmed with the treatment of ERK siRNA (Additional file 3: Figure S3B). These findings suggested that the MEK inhibitor compensated for the induced p-ERK and EGFR expression triggered by the HDAC inhibitor.

### HDAC and MEK are involved in the HDACi-MEKi-combined apoptotic effect

**Fig. 5:**
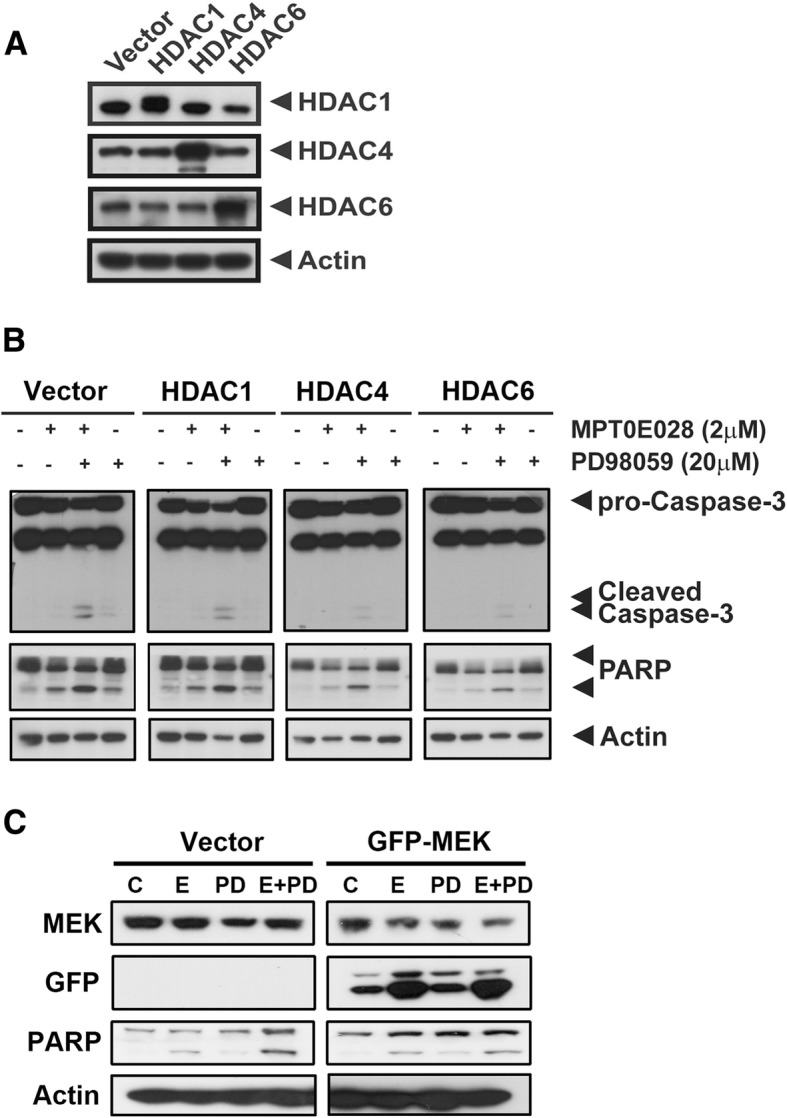
Overexpressed HDAC4, HDAC6, or MEK can partially reverse the apoptotic effect of combination. AsPC-1 cells were transfected with empty vector, HDAC1, HDAC4, HDAC6, or GFP-MEK overnight and then co-treated with 2 μM MPT0E028 and 20 μM PD98059 for 48 h. **a** The protein expression of HDAC1, HDAC4, HDAC6, **b** caspase-3, and PARP. Actin was used as an internal control. **c** MEK, GFP, and PARP expressions were determined after overexpressed MEK AsPC1 cells co-treated with MPTE028 (2 μM) and PD98059 (20 μM). C control, E MPT0E028, PD PD98059

Our previous study showed that MPT0E028 enzymatically inhibited Class I and II HDACs [15]. In addition, MPT0E028 inhibited the growth of human B cell lymphoma cells through HDAC suppression. Therefore, to further determine whether HDACs and MEK play synergistic roles in apoptosis, HDACs and MEK were transiently overexpressed in pancreatic cancer cells followed by co-treatment with MPT0E028 and PD98059. As shown, HDAC1, 4, and 6 were all transfected successfully (Fig. 5a and Additional file 4: Figure S4A), and the MPT0E028 and PD98059-induced cleavage of caspase-3 and PARP activation were abolished by HDAC4 or HDAC6 overexpression but were not affected by HDAC1 (Fig. 5b and Additional file 4: Figure S4B). Furthermore, diminished PARP activity was detected in response to MEK overexpression (Fig. 5c and Additional file 4: Figure S4B). These results suggest that HDAC and MEK proteins are involved in the MPT0E028 and PD98059-mediated apoptosis pathway activation.

### MPT0E028 and PD98059 combination shows antitumor activity in a human pancreatic AsPC-1 xenograft model

**Fig. 6:**
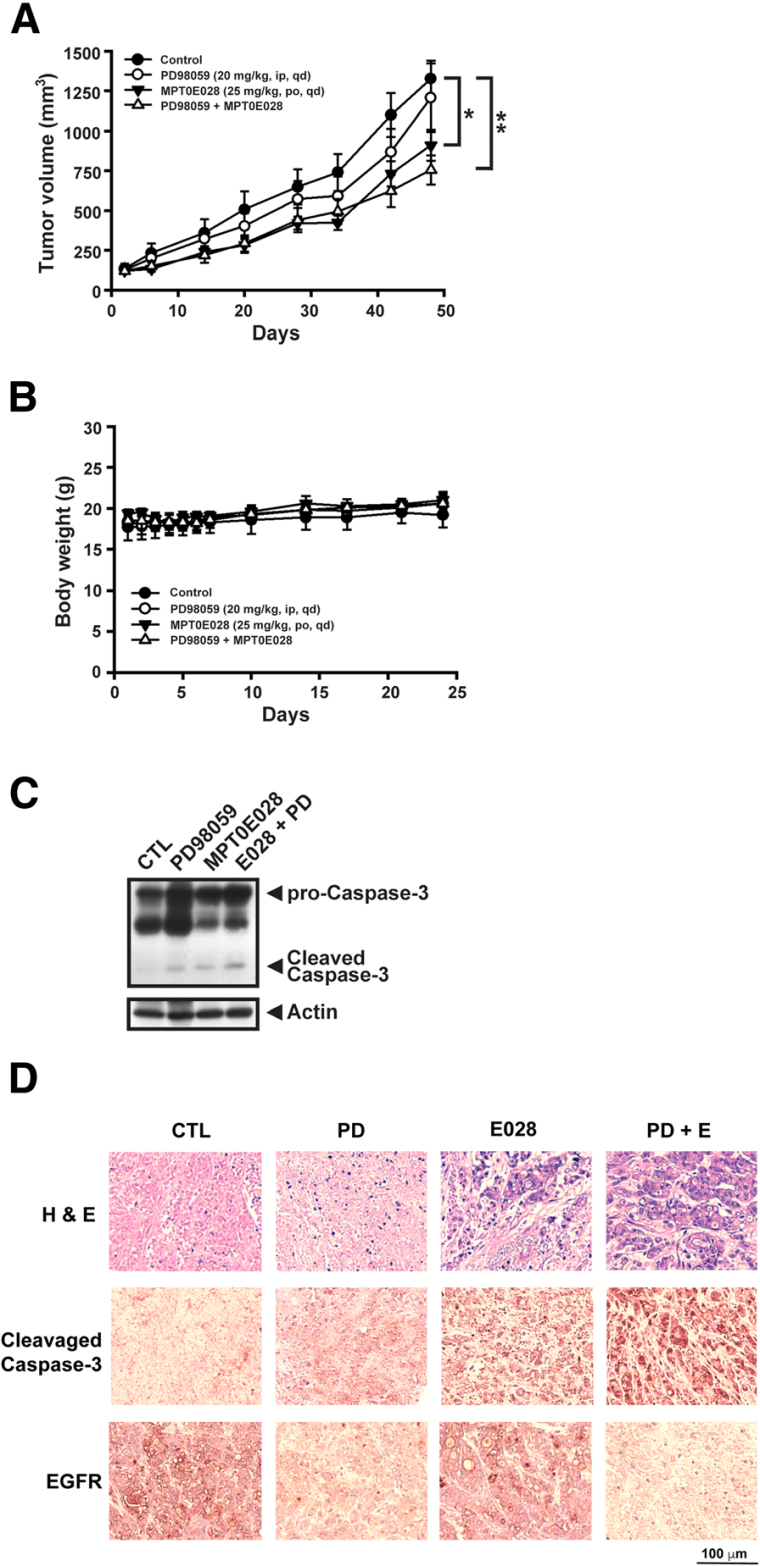
The antitumor activity of combination MPT0E028 with PD98059 in human pancreatic cancer xenograft model. AsPC-1 cells were transplanted subcutaneously in Balb/c-nude mice. Mice were randomized into four groups with daily treatment: vehicle control, MPT0E028 (oral, 25 mg/kg), PD98059 (intraperitoneal (ip), 20 mg/kg), or combinational therapy. Tumor volume of AsPC-1 xenograft was shown in **a** and the body weights were revealed in **b**. *P < 0.05 and **P < 0.01 compared with the respective control group. **c** Tumor protein lysates were subjected to immunoblot with caspase-3 antibody. Actin was served as a loading control. **d** Hematoxylin and eosin staining was performed to examine cell morphology, and the immunohistochemical staining determines the expressions of cleavage caspase-3 and EGFR before or after indicated drug treatment. PD PD98059, E MPT0E028

Finally, to investigate the in vivo antitumor activity of the proposed combinational strategy, we used AsPC-1 xenografts in a nude mouse model. Once the tumor volume reached 100 mm^3^, the mice were randomly divided into four groups: vehicle control group, PD98059 (20 mg/kg daily) alone group, MPT0E028 (25 mg/kg daily) alone group, or combinational therapy group. Compared to the control group, MPT0E028 treatment alone and the combinational therapy resulted in the reduction of the tumor volume. Further, there was a trend displayed that the combination group had an additional antitumor effect (Fig. 6a). There was no loss of body weight or adverse effects noted in any group (Fig. 6b). Immunoblot analysis of AsPC-1 xenograft tissues showed an increase in the cleaved caspase-3 level in the combination therapy group, which was confirmed with immunohistochemical staining (Fig. 6c). Moreover, EGFR expression was downregulated following co-treatment of the two drugs (Fig. 6d). These results indicated that the combination of MPT0E028 and PD98059 would have potential antitumor activity in vivo.

## Discussion

Pancreatic cancer is a lethal disease with a poor and dismal prognosis. Since curable drug treatment is currently unavailable, a novel efficacious therapeutic strategy is urgently needed. HDAC inhibitors have been demonstrated to have anticancer potential for pancreatic cancer [12]. In addition, mutated K-Ras and overexpressed EGFR is present in > 90% and 30–50% of pancreatic cancers, respectively, resulting in downstream MEK/ERK pathway overactivation [30]. Hence, we evaluated the anticancer potential and mechanism of a MEK inhibitor combined with our novel HDAC inhibitor MPT0E028, which currently completes phase I clinical trial for patients with solid tumors, against pancreatic cancer to provide a guide for a future developmental strategy.

The observation of aberrant HDAC expression in pancreatic cancer led to discovery of the therapeutic potential of HDAC inhibitors in the treatment of pancreatic ductal adenocarcinoma [31]. In preclinical studies, the mechanisms of HDAC inhibitors against pancreatic cancer cells were demonstrated to involve apoptosis activation, along with the induction of anti-angiogenesis and anti-metastasis factors [12]. MPT0E028, an orally administered *N*-hydroxyacrylamide-derived pan-HDAC inhibitor, showed a growth suppression effect in cultured NCI-60 human cancer cell lines, including leukemia, melanoma, lung, colon, breast, prostate, renal, and central nervous system cancers, in our previous study [15]. Here, we found that treatment with MPT0E028 or the commercially available pan-HDAC inhibitor SAHA alone could evoke the apoptosis of pancreatic cancer cells and accumulation of cells in the sub-G1 phase at 72 h (Fig. 3). But, compared to K-Ras codon 12 mutant (K12D) AsPC-1 and PANC-1 cells [32], K-Ras wild-type BxPC-3 was more sensitive to the HDAC inhibitors (Figs. 1, 2 and Additional file 5: Table S1). Further research is required to determine the specific causes of these differences in the sensitivity of pancreatic cells to HDAC inhibitors.

Based on completed clinical trials, the use of an HDAC inhibitor as monotherapy in hematological malignancies showed more effective therapeutic outcomes than for solid tumors; therefore, the combination of HDAC inhibitors with various anticancer agents has been evaluated for the treatment of advanced pancreatic cancer [12]. In addition, Ras has been recognized as an undruggable target in pancreatic cancers characterized by overactivation of the Ras-Raf-MEK-ERK pathway. Administration of a MEK inhibitor alone failed to provide promising antitumor effects in pancreatic cancer patients [10]. Therefore, we combined an HDAC inhibitor with a MEK inhibitor, which had a synergistic effect on decreasing cell viability, although this effect was exclusively observed in pancreatic cancer cells independent on K-Ras status (Figs. 1, 2 and Additional file 1: Figure S1). Moreover, the MEK inhibitor PD98059 potentiated the HDAC inhibitor-caused sub-G1 accumulation and the cleavage of caspase-3 and PARP in both AsPC-1 and PANC-1 cells (Fig. 3). These effects were stronger in AsPC-1 cells, consistent with the lower CI values of AsPC-1 compared to PANC-1 and BxPC-3 cells (Figs. 1 and 2). These observations are in line with previous studies demonstrating MEK inhibition sensitization of HDAC inhibitor-caused cell death in leukemia, breast, colon, and lung cancer cells through blockade of ERK signaling [17–19, 33, 34].

The improved anticancer effect of HDAC inhibitors enhanced by MEK inhibition can be achieved through diverse mechanisms such as NOXA-mediated Mcl-1 degradation in triple-negative breast cancer [33], c-FLIPL downregulation in B-Raf mutation-positive colon cancer [35], or activation of FOXOs with a subsequent increase in BIM and cell cycle inhibitors in lung cancers harboring a Ras mutation [34]. Hence, to further assess the underlying mechanism of pancreatic cancer cells that are synergistically susceptible to the combined therapy, we evaluated the effects on ERK activation. In AsPC-1 K-Ras mutation cells, low concentrations (below the half-maximal inhibitory value) of MPT0E028 or SAHA effectively decreased the p-ERK expression level in a dose-dependent manner at 24 h. However, to our surprise, long-term (72 h) treatment with low HDAC inhibitor concentrations dramatically activated ERK (Fig. 4a), which can be diminished by adding MEK inhibitors (Fig. 4b, c). Consistently, this phenomenon was also found in K-Ras wild-type cell line BxPC-1 (Fig. 4c). The finding suggested that the synergistic combination phenomenon might be due to the MEK inhibitor covering for HDAC inhibitor-induced p-ERK upregulation, accompanied by cell survival and drug resistance [36].

Liao and colleagues [37] reported that ERK phosphorylated CFL-1 conferred liver cancer cells with resistance to HDAC inhibitors. In addition, we previously found that MPT0E028 and SAHA triggered p-ERK expression with a subsequent increase in the transcription level of fibroblast growth factor receptor 3 in liver cancer cells resistant to sorafenib, an oral multiple kinase inhibitor [38]. In the present study, sole administration of HDAC inhibitors upregulated EGFR expression, which would be followed by ERK phosphorylation, and these effects were abrogated by MEK inhibitor at the transcription level (Fig. 4d, Additional file 2: Figure S2B and C). Our assumption about the sequence of p-ERK and EGFR activation was further validated given that EGFR expression was observed after ERK suppression. Therefore, pharmacological inhibition or genetic ablation of ERK effectively attenuated EGFR expression (Additional file 3: Figure S3). Indeed, oncogenic K-Ras has been shown to upregulate endogenous EGFR expression, which is required for K-Ras-induced pancreatic tumorigenesis [39]. Moreover, EGFR signaling directly mediates the phosphorylation of ERK bypassing Ras. Hence, MEK inhibition rather than inhibition of Raf or PI3K/AKT can result in complete elimination of EGFR-regulated ERK phosphorylation in pancreatic cancer cells [40]. MEK overexpression also reversed the apoptotic effect (PARP cleavage) of MPT0E028 and PD98059 co-administration (Fig. 5c and Additional file 4: Figure S4B). Given that MEK inhibition efficaciously diminished the activation of ERK and EGFR due to HDAC inhibitors, we consider there to be a synergistic interaction between MEK and HDAC inhibitors (Fig. 4).

Diverse HDACs govern cancer development and progression via histone or non-histone modulation [13]. A study of 29 patients with pancreatic adenocarcinoma and nine patients with chronic pancreatitis showed that the expression levels of HDAC1, 2, 4, and 7 were significantly increased in the former group [41]. HDAC6 has also been reported to be highly expressed in human pancreatic cancer tissues and was associated with increased cell migration [42]. Moreover, a recent study showed that the expression levels of HDAC1, 2, 4, and 6 were associated with clinicopathological parameters of pancreatic adenocarcinoma patients, including the tumor proliferative capacity and patient survival [43]. We found that HDAC4 and HDAC6, but not HDAC1, play critical roles in the anticancer effect of the MPT0E028/PD98059 combination in K-Ras mutant cells through triggering apoptosis (Fig. 5b and Additional file 4: Figure S4B).

Although targeting K-Ras signaling alone fails to evoke massive tumor cell death, which has limited its clinical use in the treatment of pancreatic cancer, the addition of HDAC inhibitors can greatly improve outcomes [44]. The present study provides the first demonstration of the synergistic mechanism of the HDAC/MEK inhibitor combination in pancreatic cancer cells. HDAC inhibitors induce cellular differentiation through histone and non-histone protein acetylation. These agents require further investigation as, although they have shown therapeutic benefit in combination for treating solid tumors, they may also have unexpected, resistance-inducing effects (such as upregulation of pERK and EGFR after a long-term treatment, which could be eliminated by co-treatment with a MEK inhibitor). Specifically, the MEK inhibitor compensated for the constitutive ERK phosphorylation and activated EGFR induced by MPT0E028, while MPT0E028 contributed to cell death.

Beyond cell death, the HDAC inhibitors contribute to cellular differentiation and inhibition of proliferation—both biological processes are tightly coordinated and regulated. Therefore, treatment with HDAC inhibitors renders cancer cells more sensitive to associated chemotherapeutic agents resulting in an additive/synergistic antitumor effect. This may be accompanied by positive changes in cellular metabolism and angiogenesis-related gene expression (decrease in VEGF expression). This class of drugs also has immunomodulatory properties. In this regard, tumor immune infiltration could be interesting to determine in response to this treatment strategy.

Finally, the results from the in vivo study show the higher (and significant) effect of MPT0E028 compared to MEK inhibitor when applied alone in reducing the tumor volume. The drugs seem to be well tolerated as there was no loss of body weight or adverse effects noted. The combination therapy showed a potential antitumor effect as demonstrated by tissue analysis and immunohistochemistry. (Fig. 6).

## Conclusions

To date, very few clinical trials have been conducted for assessing the effect of HDAC inhibitors in pancreatic cancer, which remains a challenging disease to treat. Treatment strategies of combining HDAC inhibitors with gemcitabine, radiation therapy, 5-FU, or bortezomib have thus far failed to improve the outcome [45]. Our newly developed pan-HDAC inhibitor MPT0E028, which completed phase I clinical trial, in combination with a MEK inhibitor showed a promising antitumor effect for pancreatic cancers especially with a K-Ras mutation. These preclinical results provide rational evidence for progressing to phase II trials of MPT0E028.

## Additional files

Additional file 1: **Figure S1.** Cytotoxic effect of combination HDACi with MEK inhibitors in BxPC-3 and PANC-1 cells. (A, B) BxPC-3 cells were treated with DMSO, MPT0E028 (E028)/SAHA, PD98059 (PD), or MPT0E028/SAHA plus PD98059 with indicated concentration for 72 h. (C, D) PANC-1 cells were treated with DMSO, MPT0E028/SAHA, trametinib (T), or MPT0E028/SAHA plus trametinib with indicated concentration for 72 h. Left panels: Cell viability was determined by MTT assay. Right panels: Combination index (CI) and fraction affected (Fa) are calculated by CompuSyn software. T0.1 and T1 were represented here as trametinib 0.1 μM and 1 μM. (TIF 440 kb)

Additional file 2: **Figure S2.** MEK inhibitors downregulate HDAC inhibitor-induced p-ERK and EGFR expression. (A) PANC-1 cells were treated with 5 μM MPT0E028 or SAHA combined with or without 20 μM PD98059 (PD) for 72 h. The protein expression of p-ERK and EGFR was determined by western blotting. t-ERK was used as an internal control. PANC-1 (B) and BxPC-3 (C) cells were treated with indicated conditions for 48 h to determine EGFR mRNA expression. *P < 0.05, **P < 0.005, and ****P < 0.0001 compared with the indicated group. (TIF 680 kb)

Additional file 3: **Figure S3.** ERK inhibition can downregulate EGFR expression. (A) AsPC-1 cells were treated with indicated concentrations of PD98059 for 24, 48, and 72 h. p-ERK and EGFR protein levels were determined. (B) AsPC-1 cells were transfected with scramble or ERK siRNA for 48 h to evaluate the protein expression of ERK and EGFR. Actin was loaded as an internal control. (TIF 440 kb)

Additional file 4: **Figure S4.** Overexpressed HDAC4, HDAC6, or MEK can reverse the apoptotic effect of combination in PANC-1 cells. PANC-1 cells were transfected with empty vector, HDAC1, HDAC4, HDAC6, or GFP-MEK plasmid overnight and then co-treated with 2 μM MPT0E028 and 20 μM PD98059 for 48 h. (A) The protein expression of HDAC1, HDAC4, HDAC6, GFP, and (B) PARP. Actin was used as an internal control. E, MPT0E028; PD, PD98059. (TIF 1669 kb)

Additional file 5: **Table S1.** IC_50_ of MPT0E028 and SAHA in pancreatic cell lines. AsPC-1, PANC-1, and BxPC-3 cells were treated with different concentrations of MPT0E028 or SAHA for 72 h. IC_50_ (the half maximal inhibitory concentration) was determined by MTT assay. (DOCX 12 kb)

## Abbreviations

CI: Combination index
EGFR: Epithelial growth factor receptor
HDAC: Histone deacetylase
