# Supplementary figures and images for: Combination treatment strategy for pancreatic cancer involving the novel HDAC inhibitor MPT0E028 with a MEK inhibitor beyond K-Ras status
Source: Clin Epigenetics. 2019 May 29;11:85. doi: 10.1186/s13148-019-0681-6 (PMC6540419; doi:10.1186/s13148-019-0681-6)

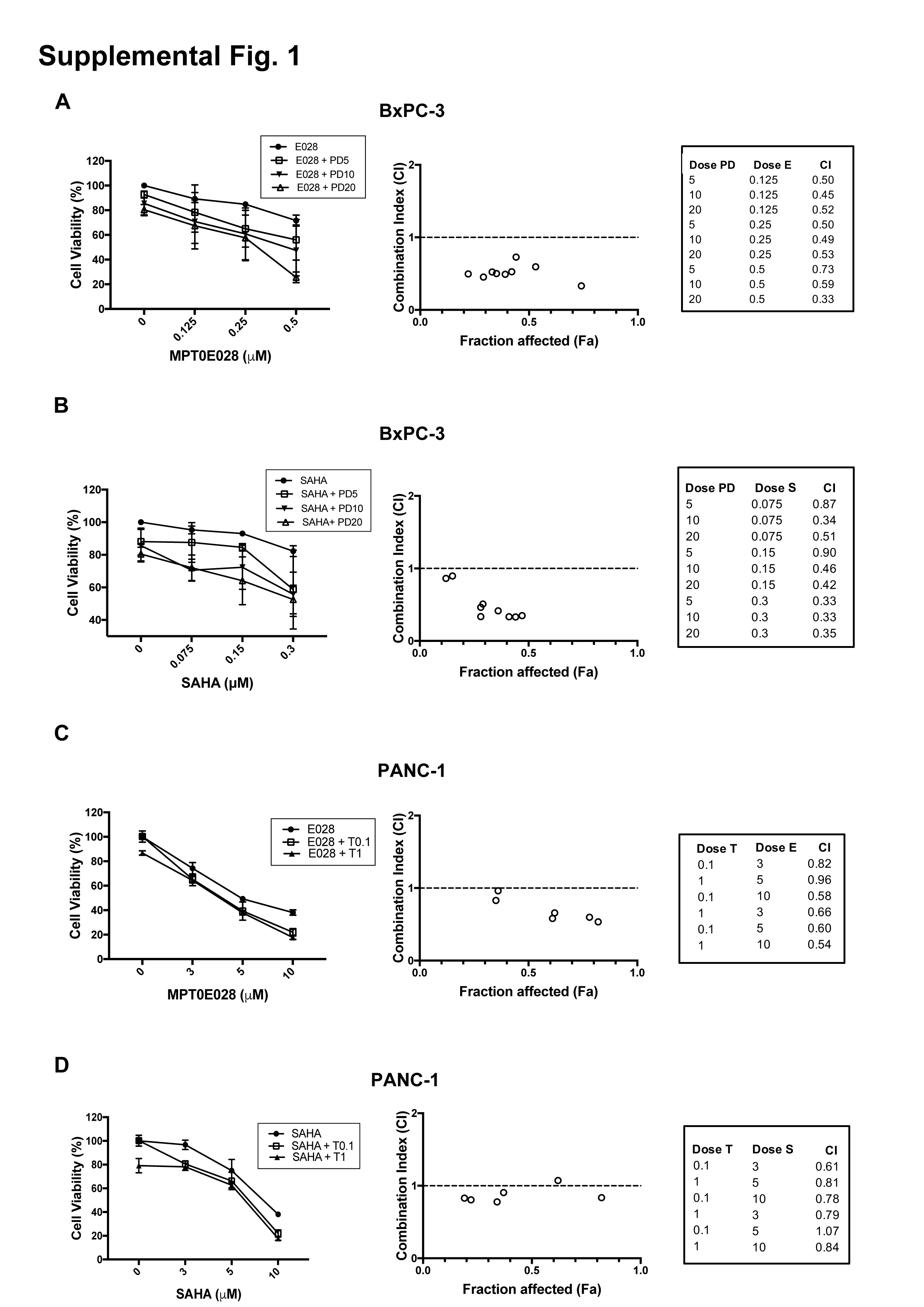

Supplement: Supplementary file 1 — Figure S1. Cytotoxic effect of combination HDACi with MEK inhibitors in BxPC-3 and PANC-1 cells. (A, B) BxPC-3 cells were treated with DMSO, MPT0E028 (E028)/SAHA, PD98059 (PD), or MPT0E028/SAHA plus PD98059 with indicated concentration for 72 h. (C, D) PANC-1 cells were treated with DMSO, MPT0E028/SAHA, trametinib (T), or MPT0E028/SAHA plus trametinib with indicated concentration for 72 h. Left panels: Cell viability was determined by MTT assay. Right panels: Combination index (CI) and fraction affected (Fa) are calculated by CompuSyn software. T0.1 and T1 were represented here as trametinib 0.1 μM and 1 μM. (TIF 440 kb) [file 13148_2019_681_MOESM1_ESM.tif]

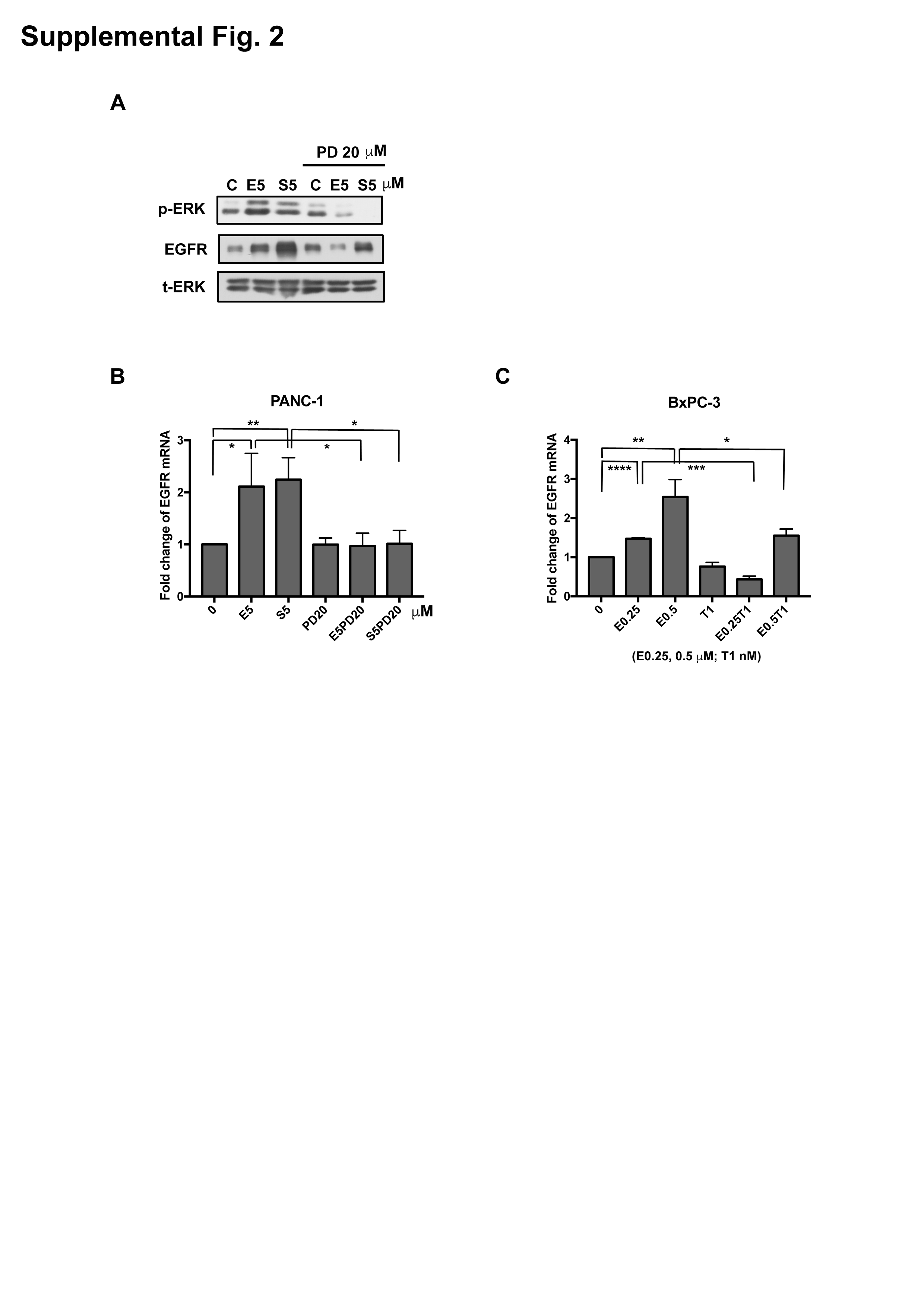

Supplement: Supplementary file 2 — Figure S2. MEK inhibitors downregulate HDAC inhibitor-induced p-ERK and EGFR expression. (A) PANC-1 cells were treated with 5 μM MPT0E028 or SAHA combined with or without 20 μM PD98059 (PD) for 72 h. The protein expression of p-ERK and EGFR was determined by western blotting. t-ERK was used as an internal control. PANC-1 (B) and BxPC-3 (C) cells were treated with indicated conditions for 48 h to determine EGFR mRNA expression. *P < 0.05, **P < 0.005, and ****P < 0.0001 compared with the indicated group. (TIF 680 kb) [file 13148_2019_681_MOESM2_ESM.tif]

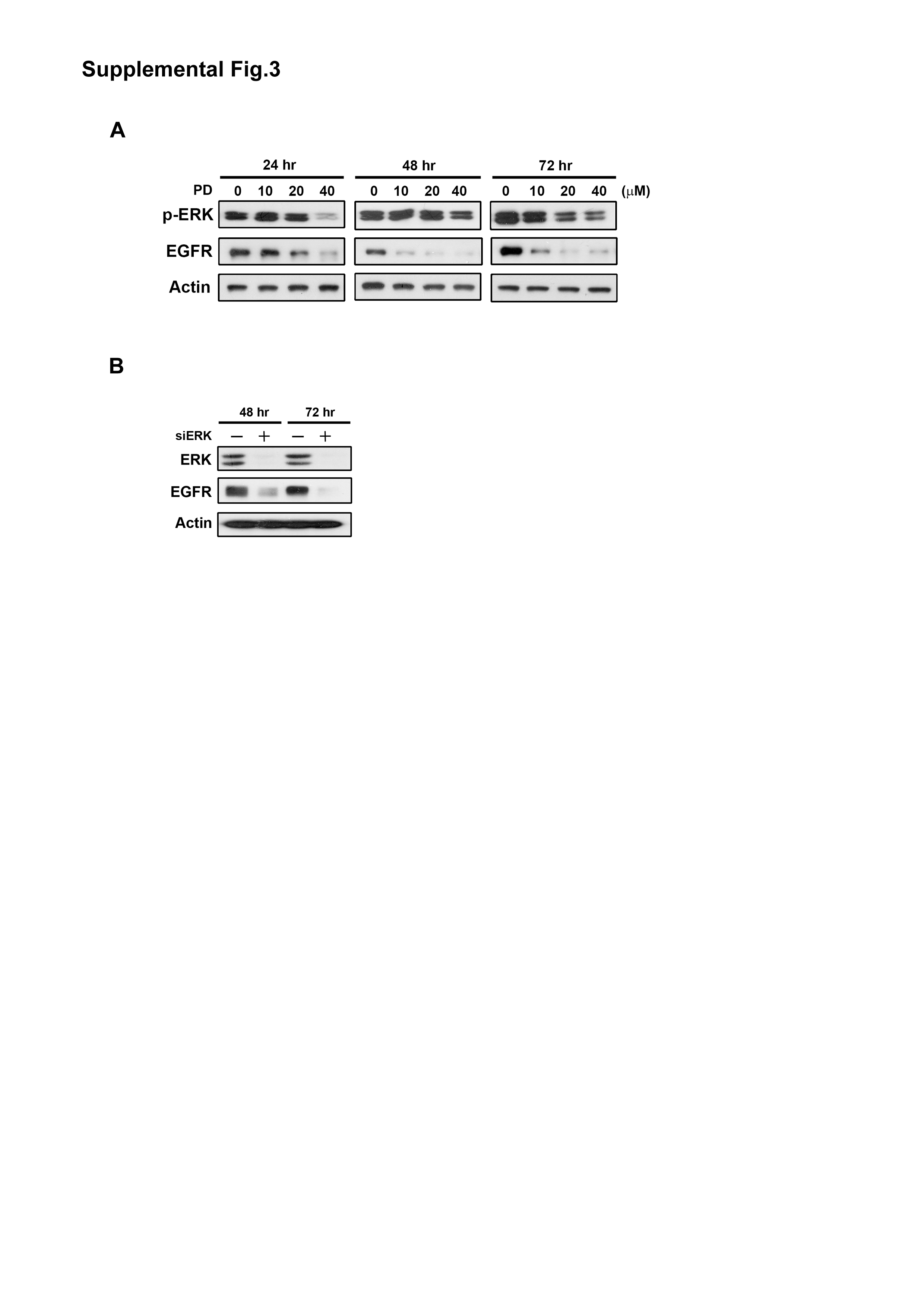

Supplement: Supplementary file 3 — Figure S3. ERK inhibition can downregulate EGFR expression. (A) AsPC-1 cells were treated with indicated concentrations of PD98059 for 24, 48, and 72 h. p-ERK and EGFR protein levels were determined. (B) AsPC-1 cells were transfected with scramble or ERK siRNA for 48 h to evaluate the protein expression of ERK and EGFR. Actin was loaded as an internal control. (TIF 440 kb) [file 13148_2019_681_MOESM3_ESM.tif]

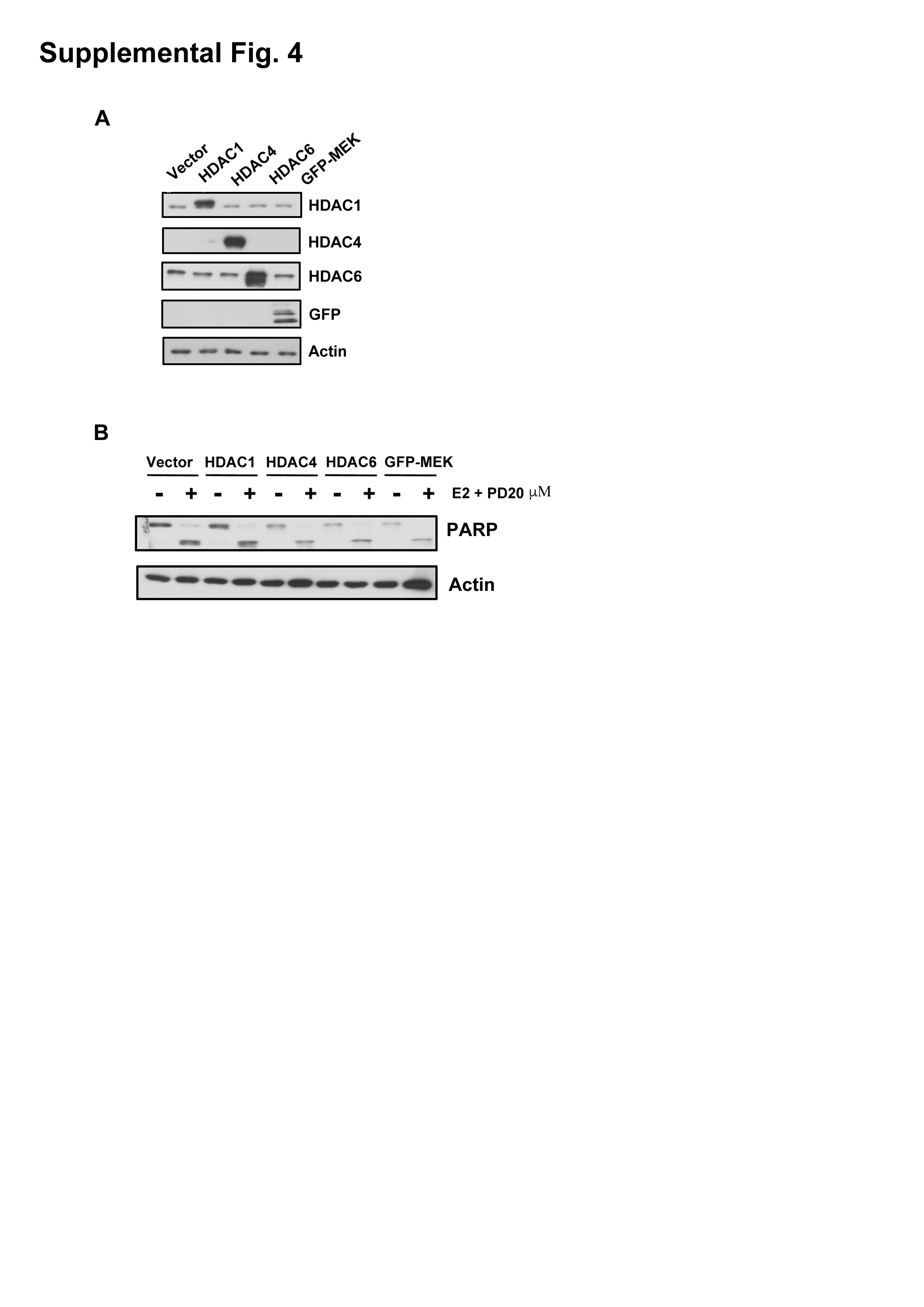

Supplement: Supplementary file 4 — Figure S4. Overexpressed HDAC4, HDAC6, or MEK can reverse the apoptotic effect of combination in PANC-1 cells. PANC-1 cells were transfected with empty vector, HDAC1, HDAC4, HDAC6, or GFP-MEK plasmid overnight and then co-treated with 2 μM MPT0E028 and 20 μM PD98059 for 48 h. (A) The protein expression of HDAC1, HDAC4, HDAC6, GFP, and (B) PARP. Actin was used as an internal control. E, MPT0E028; PD, PD98059. (TIF 1669 kb) [file 13148_2019_681_MOESM4_ESM.tif]
