# Supplementary material for: Combination treatment strategy for pancreatic cancer involving the novel HDAC inhibitor MPT0E028 with a MEK inhibitor beyond K-Ras status
Source: Clin Epigenetics. 2019 May 29;11:85. doi: 10.1186/s13148-019-0681-6 (PMC6540419; doi:10.1186/s13148-019-0681-6)
Supplement: Supplementary file 5 — Table S1. IC50 of MPT0E028 and SAHA in pancreatic cell lines. AsPC-1, PANC-1, and BxPC-3 cells were treated with different concentrations of MPT0E028 or SAHA for 72 h. IC50 (the half maximal inhibitory concentration) was determined by MTT assay. (DOCX 12 kb) [file 13148_2019_681_MOESM5_ESM.docx]

**Supplemental Table 1**. IC_50_ of MPT0E028 and SAHA in pancreatic cell lines

|  | | |
| --- | --- | --- |
|  | **MPT0E028** | **SAHA** |
| IC_50_ ± SE (μM) | | |
| **AsPC-1** | 3.73 ± 0.804 | 5.65 ± 0.441 |
| **PANC-1** | 7.9 ± 0.387 | 13.45 ± 1.5 |
| **BxPC-3** | 0.65 ± 0.082 | 2.28 ± 1.056 |
